# AL amyloidosis: The effect of fluorescent in situ hybridization abnormalities on organ involvement and survival

**DOI:** 10.1002/cam4.3683

**Published:** 2020-12-21

**Authors:** Michael Ozga, Qiuhong Zhao, Don Benson, Patrick Elder, Nita Williams, Naresh Bumma, Ashley Rosko, Maria Chaudhry, Abdullah Khan, Srinivas Devarakonda, Rami Kahwash, Ajay Vallakati, Courtney Campbell, Samir V. Parikh, Salem Almaani, Jason Prosek, Jordan Bittengle, Katherine Pfund, Samantha LoRusso, Miriam Freimer, Elyse Redder, Yvonne Efebera, Nidhi Sharma

**Affiliations:** ^1^ Division of Hematology Department. of Internal Medicine The Ohio State University Comprehensive Cancer Center Columbus OH USA; ^2^ Division of Cardiology Department of Internal Medicine The Ohio State University Columbus OH USA; ^3^ Division of Nephrology Department of Internal Medicine The Ohio State University Columbus OH USA; ^4^ Department of Neurology The Ohio State University Columbus OH USA; ^5^ Department of Oncology Rehabilitation The Ohio State University Columbus OH USA

**Keywords:** amyloidosis, fluorescent in situ hybridization, light‐chain amyloidosis, myeloma, plasma cell burden, survival

## Abstract

**Background:**

Systemic light chain (AL) amyloidosis is a clonal plasma‐cell neoplasm that carries a poor prognosis. Although AL amyloidosis and Multiple Myeloma (MM) can co‐exist and share various cytogenetic chromosomal abnormalities, little is known about Fluorescent in situ hybridization (FISH) and its prognostic relevance in AL amyloidosis.

**Aim:**

The study aims to evaluate the most prevalent FISH cytogenetic abnormalities in AL patients as independent prognostic factors, and assess the impact of cytogenetics on the survival of high‐risk cardiac AL patients.

**Materials & Methods:**

This retrospective study reviewed 113 consecutive AL patients treated at The Ohio State University (OSU). Patients were divided into subgroups based on FISH data obtained within 90 days of diagnosis. Hyperdiploidy was defined as trisomies of at least 2 chromosomal loci. Primary endpoints were progression free survival (PFS) and overall survival (OS). Kaplan Meier curves were used to calculate PFS and OS. The log‐rank test and Cox proportional hazard models were used to test the equality of survival functions and further evaluate the differences between groups.

**Results:**

FISH abnormalities were detected in 76% of patients. Patients with abnormal FISH trended toward lower overall survival (OS) (p=0.06) and progression free survival (PFS) (p=0.06). The two most prevalent aberrations were translocation t(11;14) (39%) and hyperdiploidy‐overall (38%). Hyperdiploidy‐overall was associated with worsening PFS (p=0.018) and OS (p=0.03), confirmed in multivariable analysis. Patients with del 13q most frequently had cardiac involvement (p=0.006) and was associated with increased bone marrow plasmacytosis (p=0.02). Cardiac AL patients with no FISH abnormalities had much improved OS (p=0.012) and PFS (p=0.018)

**Conclusions:**

Our findings ultimately reveal the association of hyperdiploidy on survival in AL amyloidosis patients, including the high‐risk cardiac AL population.

## Graphical abstract

Hyperdiploidy in AL amyloidosis patients was associated with poor PFS and OS and cardiac AL patients had a strong association with del 13q.

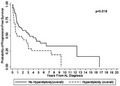

## 1 INTRODUCTION

Systemic light chain (AL) amyloidosis is a clonal plasma‐cell neoplasm that confers a poor prognosis. AL amyloidosis is characterized by production of misfolded light chains that subsequently deposit in key organs as amyloid fibrils.^1^, ^2^ AL amyloidosis is typically diagnosed at an advanced stage when treatment options are limited and do little in changing its course. Efforts are being made at recognizing this disease early and developing better prognostic tools. Cytogenetic analysis and its prognostic significance have been well studied in multiple myeloma (MM), a related disorder, but its utility remains relatively unknown in AL amyloidosis.^3^, ^4^ A study conducted by Bryce et al^5^ in 2009 was one of the first to describe the utility of interphase fluorescence *in situ* hybridization (FISH) coupled to cytoplasmic staining of specific IgH (cIg‐FISH) on bone marrow plasma cells, specifically identifying t(11;14) as an adverse risk factor in AL patients. Warsame et al^6^ also reported on cIg‐FISH abnormalities, analyzing degree of plasma cell burden and their relationship to survival and advanced cardiac disease. Muchtar et al^7^ further stratified interphase FISH cytogenetic AL amyloidosis results and showed t(11;14) positive patients who received bortezomib and immunomodulatory (IMiD)‐based regimens had inferior survival compared with those lacking t(11;14). They also showed trisomies to be associated with a shorter overall survival. Various other studies have investigated specific FISH probes and their corresponding cytogenetics abnormalities, including 1q21 gain, 17p deletion, various trisomies (chromosomes 3,7,9,15), and hyperdiploidy.^4^, ^5^, ^6^, ^7^, ^8^, ^9^, ^10^ This will likely continue to grow as there continues to be an increase in the variety of FISH probe analyses available.

Expanding on the impact of cytogenetics on AL amyloidosis survival, the aim of this study was to identify the most relevant FISH biomarkers present in our AL amyloidosis patient subset and establish their importance as independent prognostic factors for survival. We also assessed the impact of chromosomal abnormalities on the survival of high‐risk cardiac AL amyloidosis patients all in an effort to ultimately provide additional prognostic information in these often‐complex patients.

## 2 PATIENTS AND METHODS

### 2.1 Patient population

We performed a retrospective chart review on 113 AL patients treated at The Ohio State University between 2001 and 2019. The study was approved by our institutional review board and follows the principles of the Declaration of Helsinki. All patients had FISH performed as a routine clinical test within 90 days of diagnosis. Additionally, 18 of these patients formed a smaller cohort who received daratumumab during their treatment course.

### 2.2 FISH studies

CD138‐enriched chromosome‐specific FISH panels for MM and cytogenetics obtained from bone marrow aspirate samples were studied, with most samples collected at our institution. FISH enumeration strategies were utilized to detect monosomies (deletions) or gains (trisomies/tetrasomies) of the following chromosomes with respective probe sets: 17 (17p13.1 and 17q21), 13 (13q14 RB1 and 13q34 LAMP1), 3 (D3Z1), 6 (6q21), 5 (5q33‐34,5p152(CSF1R‐D5S23:D5S721)), 19 (19p13 TCF), 12 (12p13 ETV6 and 12cen), 1 (both 1q21 CKS1B/1q23 PBX1 and 1p CHD5), 11 (both 11q13 CCND1 and 11q23 ATM), 9 (D9Z1), and 15 (D15Z4). We also analyzed translocations involving the immunoglobulin heavy chain (IgH) and several partners, most notably 11q13 (CCND1), followed by 4p16.3 (FGFR3), 16q23 (MAF), 20q12 (MAFB), and 6p21 (CCND3). Patients were divided into subgroups based on the above FISH data, including a binary assessment of abnormal (presence of any abnormality) versus normal FISH samples. Furthermore, the criteria established by Wuilleme et al^10^ regarding the definition of hyperdiploidy, which requires trisomies of at least two or more of the three chromosomes 5, 9, and 15, was globally expanded to include two or more trisomies/gains of any chromosomal loci, henceforth, referred to as “hyperdiploidy‐overall.” We also selectively analyzed gains of 1q21, 5p/5q, and 11q23 to create a “hyperdiploidy high‐risk” group that included two or more of these specific trisomies, with the idea of co‐segregating adverse FISH markers initially proposed in MM analysis by Boyd et al.^11^

### 2.3 Data collection

All laboratory values were collected at the time of diagnosis and recorded in the electronic medical record. Bone marrow clonal plasma cells (PCs) were grouped into <10% and ≥10%, with ≤10% PCs indicating coexistent monoclonal gammopathy of undetermined significance (AL+MGUS), and ≥10% PCs indicating smoldering multiple myeloma (AL+SMM) or multiple myeloma (AL+MM) based on International Myeloma Working Group consensus criteria.^12^ Kappa (κ) and lambda (λ) light chain restriction was recorded, as well as the resultant κ/ λ ratio and difference between involved and uninvolved light chains (dFLC). Organ involvement was recorded, delineated into “cardiac,” “kidney,” “hepatic,” “gastrointestinal,” “peripheral neuropathy,” or “other” (i.e. soft tissue), based on tissue biopsies demonstrating apple green birefringence under polarized light. Related serum biomarkers and imaging studies as previously established via consensus from the International Society of Amyloidosis^1^, ^13^ was also recorded.

### 2.4 Disease staging

AL amyloidosis staging systems are primarily based on guidelines presented by the Mayo Clinic group who first established a biomarker staging system in 2004^14^ with update in 2012^15^, ^16^ that reflected circulating markers of cardiac, renal, and clonal disease. These staging systems incorporate *N*‐terminal pro‐brain natriuretic peptide (NT‐proBNP), cardiac markers (cardiac troponin T TnT or cardiac troponin I TnI), and dFLCs. The Boston Medical center (BMC) also developed a comparative AL staging system that incorporates BNP to mirror the Mayo 2004 staging. As a result, we obtained the above serum biomarkers and staged patients according to Mayo 2012, and BMC systems. Patients were classified as having stage I, II, III, or IV (if applicable) based on whether they had zero, 1, 2, or 3 risk factors.

### 2.5 Statistical analysis

Patient characteristics were summarized using median and range for continuous variables, and frequency and percentage for categorical variables. The comparison of patient characteristics between groups were conducted using Wilcoxon rank‐sum (Mann–Whitney) test for continuous variables and Fisher's exact test for categorical variables. Primary endpoints were progression free survival (PFS) and overall survival (OS), per updated National Comprehensive Cancer Network (NCCN) guidelines,^2^ including both organ and hematologic criteria. PFS was defined as the time from date of AL diagnosis to date of progression or death from any cause, censoring those who did not progress at the last clinical assessment dates. OS was defined as the time from date of AL diagnosis to death from any cause, censoring those who were still alive at the date of last follow up. Kaplan–Meier survival function was used to estimate the probability of PFS and OS, and log‐rank tests were used to evaluate the equality of survivor functions between different groups of patients. Cox proportional hazard model was used to evaluate the association between patient characteristics and risk of relapse/death. Univariable modeling was performed first for evaluation of association between each individual variable and the outcome. Variables with *p* < 0.10 in the univariable analysis were further evaluated in multivariable modeling. Using backward selection, variables that reached statistical significance remained in the final model. The significance level was set at *α* = 0.05 and all *p*‐values presented are from two‐sided tests. The statistical analysis was performed using Stata 14.

## 3 RESULTS

### 3.1 Patient characteristics

Baseline demographics and clinical characteristics of the 113 patients are shown in Table 1. For the 113 patients, the median age at diagnosis was 62 years (range: 33–84) and 58% were male. The number of patients with concomitant MGUS, SMM and MM at the diagnosis of AL amyloidosis was 52 (42.3%), 36 (29.3%) and 25 (20.3%), respectively. The majority of patients (*N* = 85, 75%) had lambda (λ) light chain (LLC) clonal disease. Abnormal FISH results were detected in 86 (76%) patients. The median number of organs involved was 2 (range 0–5), with 51% and 66% having cardiac and kidney involvement, respectively. There were 61 (54%) patients with a bone marrow plasmacytosis >= 10%. The number of patients with abnormal cytogenetics had significantly higher PC compared to patients with normal cytogenetics (59% vs. 37%, *p* = 0.043), and 25 (20.3%) patients met the criteria for concomitant MM.^12^ There were 45 patients who underwent autologous stem cell transplantation (ASCT). The majority of the patients (62%, *N* = 70) received bortezomib‐based regimens mostly in combination with cyclophosphamide.

**TABLE 1:** Patient demographics and disease characteristics at diagnosis

Characteristics	All patients (*n* = 113)	Patients with normal FISH (*n* = 27)	Patients with abnormal FISH (*n* = 86)
Median age at diagnosis (range), years	62 (33–84)	60 (36–84)	63 (37–84)
Gender, female, *n* (%)	47 (41.6)	10 (37)	37 (43.0)
Received ASCT, *n* (%)	45 (39.8)	15 (55.6)	30 (34.9)
Light chain restriction (kappa)	27 (23.9)	6 (22.2)	21 (24.4)
Light chain restriction (lambda)	85 (75.2)	20 (74.1)	65 (75.6)
dFLC mg/dl^a^	35.3 (5435–7000)	20.4 (1246–345)	51.4 (5435–7000)
BM PC^b^			
<10%	52 (46.0)	17 (63.0)	35 (40.7)
≥10%	61 (54.0)	10 (37.0)	51 (59.3)
AL + MGUS	52 (42.3)	17 (63.0)	35 (40.7)
AL + SMM	36 (29.3)	9 (33.3)	27 (31.4)
AL + MM^b^	25 (20.3)	1 (3.7)	24 (27.9)
Urine total protein, mg/24 h	1755 (0–81921)	7172 (0–81921)	1706 (0–22500)
No. of involved organs, median (range)	2 (0–5)	2 (0–4)	2 (1–5)
Cardiac involvement present, n (%)	58 (51.3)	11 (40.7)	47 (54.7)
Renal involvement present, *n* (%)	75 (66.4)	16 (59.3)	59 (68.6)
NT‐proBNP ≥332 ng/L, *n* (%)^b^	49 (80.3)	8 (66.7)	41 (83.7)
NT‐proBNP ≥1800 ng/L, *n* (%)	33 (54.1)	6 (50.0)	27 (55.1)
Alkaline phosphatase, median (range)	76 (28–472)	83 (28–275)	76 (40–472)
Mayo stage (2012), *n*(%)^c^			
I	19 (31.7)	4 (33.3)	15 (31.3)
II	16 (26.7)	5 (41.7)	11 (22.9)
III	13 (21.7)	2 (16.7)	11 (22.9)
IV	12 (20.0)	1 (8.3)	11 (22.9)
Missing	53	15	38
BMC stage (2019), *n*(%)^b^, ^d^			
I	30 (26.5)	13 (48.1)	17 (19.8)
II	44 (38.9)	9 (33.3)	35 (40.7)
III	39 (34.5)	5 (18.5)	34 (39.5)
Total number of lines of therapy received, average (range)	1.86 (1–8)	1.83 (1–7)	1.89 (1–8)
Best hematological response achieved, *n*(%)			
CR/VGPR rate	60 (53)	15 (56)	45 (52)
SD rate	37 (33)	7 (26)	30 (35)

ASCT, autologous stem cell transplant; BM, bone marrow; PC, plasma cells; FISH, fluorescent *in situ* hybridization; dFLC, difference between involved and uninvolved free light chains; MGUS, monoclonal gammopathy of undetermined significance; SMM, smoldering multiple myeloma; MM, multiple myeloma.

^a^ Kappa nl 3.3–19.4 mg/L, Lambda nl 5.71–26.3 mg/L.

^b^ *p* < 0.05.

^c^ Stage I: none the following are elevated: troponin T ⩾ 0.025 ng/ml and NT‐ProBNP ⩾ 1800 pg/ml and serum immunoglobulin free light chain difference ⩾ 18 mg/dl; if any one parameter is high, then, Stage II; if two parameters are high, then, Stage III; and if all three are elevated, then, Stage IV;

^d^ Stage I neither troponin I ≥ 0.10 or BNP ≥ 81 pg/ml; if one elevated, then, Stage II; and if both are elevated, then, Stage III.

### 3.2 FISH abnormalities and their relationship to disease burden and bone marrow plasmacytosis

**FIGURE 1:**
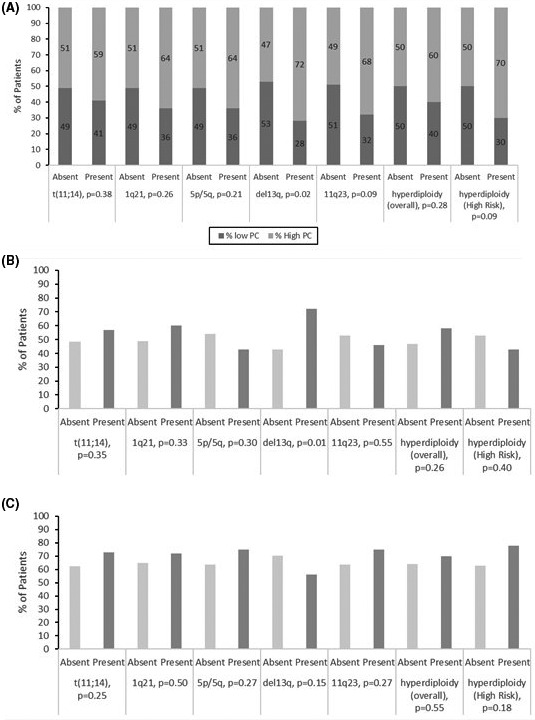
Relationship between FISH probes and bone barrow plasmacytosis, organ involvement, a) Association between FISH abnormalities and low (<10%) and high (>=10%) plasmacytosis, (b) Cardiac involvement and (c) Renal involvement

Translocation t(11;14) (39%) and hyperdiploidy‐overall (38%) were the most prevalent aberrations among patients with abnormal FISH results (Table S1).The most common trisomies observed were trisomy 5p15/5q33 (n = 25%), 11 (+11q23) (25%), and 1q21 (22%). Deletion 13q by FISH was seen in 28% of patients and only one patient expressed deletion 17p. Regarding the remaining established high‐risk FISH abnormalities seen in MM, we observed a paucity of t(4;14)‐ 0 cases, t(14;16)‐ 2 cases, and t(14;20)‐ 1 case. There were 43 patients (38%) who expressed hyperdiploidy‐overall with gains of two or more chromosomal loci, and also displayed a higher rate of distribution among concomitant MM patients (*p* < 0.001) (Table S1). Moreover, given the high frequencies of gains 5p15/5q33, 1q21, 11q23, we created a more specific hyperdiploidy‐ high risk grouping that expressed at least two of these three trisomies. This was identified in 20% of patients and was associated with a higher rate of concomitant MM (*p* < 0.001), (Table S1). While considering individual types of FISH abnormalities, the presence of deletion 13q was the only abnormality that showed association with increased bone marrow plasma cell burden (i.e. BMPC ≥ 10%) (*p* = 0.02) (Figure 1A).

### 3.3 FISH abnormalities and their relationship to organ involvement at diagnosis

We next attempted to better delineate the relationship between several FISH abnormalities and organ involvement at diagnosis, specifically analyzing cardiac and renal AL amyloidosis patients. Monosomy (del) 13q was found to be associated with cardiac involvement (*p* = 0.01), (Figure 1B). In fact, among cardiac AL amyloidosis patients, 14 were positive for del13q and had associated elevated NT‐proBNP levels. Conversely, no abnormal FISH probes correlated with renal involvement at diagnosis (Figure 1C).

### 3.4 FISH abnormalities and their relationship to survival and best hematological response achieved

After a median follow up of 4 years (range 0.9–18.2 years), the median PFS and OS from diagnosis in our total patient cohort was 2.7 (95% CI: 1.1–3.7) years and 6.1 (95% CI: 3.4–8.3) years, respectively.

**FIGURE 2:**
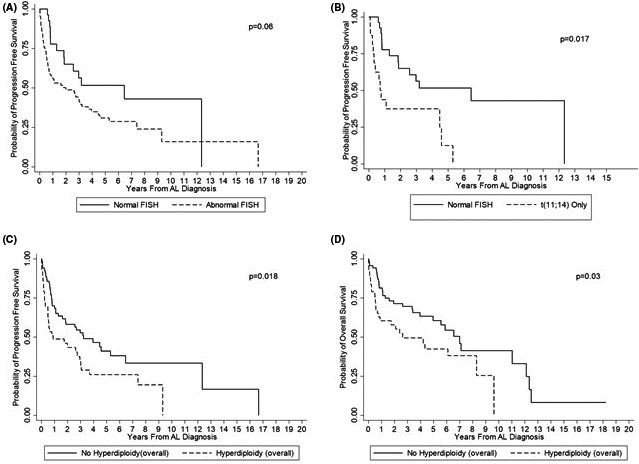
Kaplan–Meier survival estimates based on FISH abnormalities. (a) Progression‐free survival based on the presence or absence of any FISH abnormality. (b) Progression‐free survival comparing presence t(11;14) to normal FISH. (c) Progression‐free survival by presence or absence of hyperdiploidy. (d) Overall survival by presence or absence of hyperdiploidy

The median PFS was 6.5 years (95% CI: 1.8‐NR) and 2.0 years (95% CI: 0.7–3.2 years) for patients with normal and abnormal FISH, respectively, (*p* = 0.06, Figure 2a). The median OS for patients with normal FISH was 11.0 years (95% CI: 5.0‐NR) and 4.3 years (95% CI: 2.4–7.0 years) for patients with abnormal FISH, (*p* = 0.06, data not shown). When analyzing best hematological response achieved, patients with normal FISH obtained a CR/VGPR rate of 56% (15/27), compared to 52% (45/86) in the abnormal FISH grouping (*p* = 0.72). Of note, 26% (7/27) of patients with normal FISH obtained a best hematological response of stable disease (SD), compared to 35% (30/86) SD in the abnormal FISH grouping (*p* = 0.39).

More specifically, the overall presence of t(11;14) did not have any prognostic impact on OS (*p* = 0.73) or PFS (*p* = 0.48) compared to absence of t(11;14). However, upon further stratification, among four cytogenetic groups (i.e. normal FISH, t(11;14) only, t(11;14) + other abnormalities, and all other abnormalities without t(11;14)), the presence of only t(11;14) did show inferior PFS when compared to those patients with normal FISH (*p* = 0.017) (Figure 2B). This association persisted in the multivariable analysis, in which t(11;14) was the sole abnormality that was independently associated with survival after adjusting for age, cardiac and renal involvement (*p* = 0.02).

Gains of two or more chromosomal loci (hyperdiploidy‐overall) was also associated with worsening PFS (*p* = 0.018) and OS (*p* = 0.03) (Figure 2C and D), with a marginal effect of the hyperdiploidy‐high risk group on PFS (*p* = 0.07) and OS (*p* = 0.08). After adjusting for prognostic factors such as age, and cardiac involvement, hyperdiploidy‐overall and hyperdiploidy‐high risk persisted as independent negative prognostic factors (Tables S2 and S3).

**FIGURE 3:**
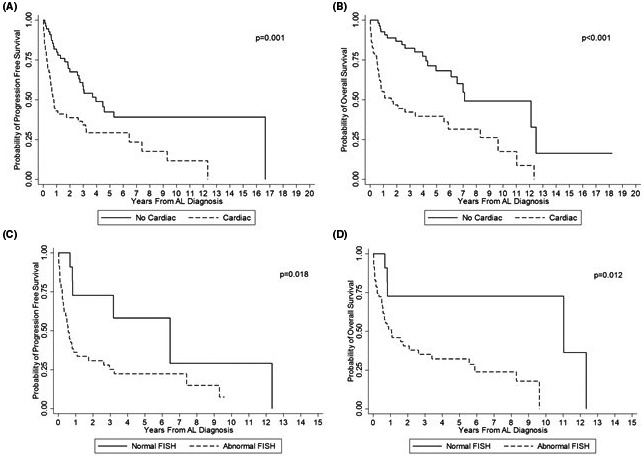
Kaplan–Meier curves demonstrating survival from diagnosis based on the presence or absence of cardiac involvement/FISH. (a) Progression‐free survival of patients with or without cardiac involvement. (b) Overall survival of patients with or without cardiac involvement. (c) Progression‐free survival among cardiac patients with or without normal FISH. (d) Overall survival among cardiac patients with or without normal FISH

With regards to organ involvement, there were 58 (51%) and 75 (66%) patients with cardiac and renal involvement, respectively. Cardiac AL patients did worse compared to their non‐cardiac AL amyloidosis counterparts, with PFS (*p* = 0.001) and OS (*p* < 0.001) (Figure 3A and B). Cardiac AL amyloidosis patients with no FISH abnormalities at diagnosis had much improved OS (*p* = 0.012) and PFS (0.018) (Figure 3C and D) compared to cardiac AL amyloidosis patients with abnormal FISH. When viewed as an isolated abnormality, cardiac patients with del13q had no significance difference in survival compared to cardiac patients without del13q. There was no difference in survival for patients with renal involvement compared to patients without renal involvement. When specifically analyzing end‐organ progression and its associated events (i.e. progression to ESRD, end‐stage CHF, or occurrence of sudden cardiac death), this was observed in 22% (6/27) of patients with normal FISH compared to 27% (23/86) in patients with abnormal FISH (*p* = 0.61).

### 3.5 FISH abnormalities and their relationship to daratumumab treated patients

Within our patient cohort (*N* = 113), 18 patients received daratumumab during their treatment course, either upfront with induction therapy and/or throughout their course in the relapsed/refractory setting. From frontline therapy initiation, we observed a median OS of 6.1 years and median PFS 2.6 years among these patients. On evaluating patients’ hematologic responses to daratumumab, there was an association between gain +1q21 and a trend toward better hematologic response. Albeit a small sample size, we observed 100% of patients (5/5) with +1q21 achieved a hematologic partial response (PR) or better when exposed to daratumumab, compared to only 54% of patients who received daratumumab without +1q21 (*p* = 0.11). Furthermore, when assessed by several different FISH parameters at diagnosis, including the presence/absence of normal FISH (*p* = 0.57), presence/absence of t(11;14) (*p* = 0.64), presence/absence of hyperdiploidy‐overall (*p* = 0.99), organ involvement at diagnosis, with/without cardiac (*p* = 0.15) or renal involvement (*p* = 0.25), patients exposed to daratumumab faired no different in terms of hematologic response (data not shown).

## 4 DISCUSSION

This comprehensive study aimed to provide a better understanding of the often‐complex behavior of aberrant plasma cells in systemic AL amyloidosis disease. In our cohort of 113 patients with FISH data at diagnosis, 86 individuals harbored abnormalities resulting in poor OS and PFS as noted above. Several studies have established worsened OS in those patients with any abnormal FISH.^5^, ^6^ Given the growing plethora of FISH probes in diagnostic evaluation, there may exist a balance between unforeseen favorable and unfavorable FISH probes that merits further exploration. Our research suggests that when data were analyzed to determine the prognostic impact of the most prevalent chromosomal aberrations, patients with the presence of t(11;14) had poor PFS compared to patients with normal FISH (*p* = 0.021). This was similar to a study by Bryce et al.^5^ This potentially suggests that these patients are at risk for early progression and may benefit from earlier upfront therapies with melphalan and/or ASCT as proposed by Muchtar et al.^7^ Moreover, despite the link observed between del13q in our study and cardiac involvement in systemic AL amyloidosis disease, we did not see a survival impact when isolating del13q as an independent prognostic marker. This was surprising given the known strong association between cardiac involvement at diagnosis and worsened AL amyloidosis staging.

In view of the low frequency of “classically‐defined” hyperdiploidy as described by Wuilleme et. al^10^ observed in our patient set (1.8%) and the paralleled low frequency observed in previous studies^6^, ^17^, ^18^, ^19^, ^20^, ^21^(range: 11–14%), we decided to broaden our definition of hyperdiploidy for this study by including gains/trisomies of 2 or more chromosomal loci (hyperdiploidy‐overall). Additionally, with the previous study by Bochtler et al^17^ and notion of co‐segregating high‐risk FISH probes in groupings proposed by Boyd et al,^11^ we decided to create our own set of high‐risk FISH probes to include gains of two or more of three probes‐5p/5q, 1q21, and 11q23 (hyperdiploidy‐high risk). This broadened definition of hyperdiploidy proved significant in survival analysis. Our hyperdiploidy and subsequent high‐risk group had poor OS and PFS and maintained its independent prognostic significance in multivariable analysis. This becomes increasingly important given the recently reported favorable prognosis of trisomies in MM,^22^ as well as the recent introduction of the probe 1q21 into routine FISH testing panels. This also potentially suggests that this high‐risk grouping may provide more utility than the previously established hyperdiploidy definition in AL amyloidosis clinical practice.

Our study also confirmed a higher level of bone marrow plasma cells in patients with del13q. As such, it is possible that those patients with del13q have a higher proclivity for amyloid deposition in organs. This aligns well with the association of del13q and cardiac involvement. We did not observe, however, a significant relationship between gain (+) 1q21 (22% of our patient cohort) and increasing bone marrow plasmacytosis. This is interesting, given that +1q21 is a known progression and risk factor in MM.^20^ We did note an interesting relationship between those patients with trisomy 1q and their hematologic response to the novel monoclonal antibody daratumumab. This anti‐CD38‐directed monoclonal antibody is approved for the treatment of multiple myeloma, but has shown promise in the treatment of relapsed/refractory AL amyloidosis patients as well. In our study, we confirmed similar promising results with 100% of patients (5/5) with +1q21 achieving a hematologic partial response or better when exposed to daratumumab. While we acknowledge very few numbers in this setting, we appreciated a possible link between +1q21 and patients’ hematologic responsiveness to daratumumab. This is also intriguing given the study done by Bochtler et al^8^ who observed an inferior survival in melphalan‐treated AL amyloidosis patients with +1q21. Routine FISH testing for 1q21 is now becoming routine practice for several institutions and identification of the mechanism by which this mutation is abrogated by daratumumab and other treatment regimens merits further exploration as its use only continues to grow.

This study has several other important limitations, largely owing to the retrospective nature of its analysis and the rare nature of this disease. We attempted to mitigate any selection bias by including all sequential patients treated at our center. Our daratumumab‐treated patient cohort was limited by daratumumab's utility in treating systemic AL amyloidosis to date, which has only recently been expanded due to the Andromeda study recently presented,^23^ and continues to grow in both upfront induction and relapsed/refractory settings.

## CONFLICT OF INTEREST

The authors declare no competing financial interests.

## Supporting information

Table S1‐S3

## Data Availability

The data that support the findings of this study are available on request from the corresponding author. The data are not publicly available due to privacy or ethical restrictions.
