# Supplementary material for: AL amyloidosis: The effect of fluorescent in situ hybridization abnormalities on organ involvement and survival
Source: Cancer Med. 2020 Dec 21;10(3):965–73. doi: 10.1002/cam4.3683 (PMC7897960; doi:10.1002/cam4.3683)
Supplement: Supplementary file 1 — Table S1‐S3 [file CAM4-10-965-s001.docx]

**Supplementary Tables**

**Supplementary Table 1. Frequency of chromosomal abnormalities in AL patients stratified by disease groups**

|  | AL+MGUS | | AL+SMM | | AL+MM | | p-value |
| --- | --- | --- | --- | --- | --- | --- | --- |
|  | n | % | n | % | n | % |  |
| t(11;14) |  |  |  |  |  |  | 0.713 |
| negative | 33 | 63.5 | 20 | 55.6 | 16 | 64.0 |  |
| positive | 19 | 36.5 | 16 | 44.4 | 9 | 36.0 |  |
| gain-1q21 |  |  |  |  |  |  | 0.125 |
| negative | 44 | 84.6 | 28 | 77.8 | 16 | 64.0 |  |
| positive | 8 | 15.4 | 8 | 22.2 | 9 | 36.0 |  |
| 5p/5q |  |  |  |  |  |  | **0.009** |
| negative | 43 | 82.7 | 29 | 80.6 | 13 | 52.0 |  |
| positive | 9 | 17.3 | 7 | 19.4 | 12 | 48.0 |  |
| del13q |  |  |  |  |  |  | **0.015** |
| negative | 44 | 84.6 | 23 | 63.9 | 14 | 56.0 |  |
| positive | 8 | 15.4 | 13 | 36.1 | 11 | 44.0 |  |
| 11q23 |  |  |  |  |  |  | **<0.001** |
| negative | 43 | 82.7 | 31 | 86.1 | 11 | 44.0 |  |
| positive | 9 | 17.3 | 5 | 13.9 | 14 | 56.0 |  |
| del17p |  |  |  |  |  |  | 0.340 |
| negative | 52 | 100.0 | 35 | 97.2 | 25 | 100.0 |  |
| positive | 0 | 0.0 | 1 | 2.8 | 0 | 0.0 |  |
| hyperdiploidy (over all probes) |  |  |  |  |  |  | **<0.001** |
| negative | 36 | 69.2 | 27 | 75.0 | 7 | 28.0 |  |
| positive | 16 | 30.8 | 9 | 25.0 | 18 | 72.0 |  |
| hyperdiploidy (High Risk probes) |  |  |  |  |  |  | **<0.001** |
| negative | 46 | 88.5 | 31 | 86.1 | 13 | 52.0 |  |
| positive | 6 | 11.5 | 5 | 13.9 | 12 | 48.0 |  |

Supplementary Table 1 Abbreviations: MGUS, monoclonal gammopathy of undetermined significance; SMM, smoldering multiple myeloma; MM, multiple myeloma. ^a^*P* < 0.05; ^b^defined as presence of 2 or more trisomies/gains of any chromosomal loci; ^c^high risk grouping composed of gains of 5p15/5q33, 1q21, 11q23.

**Supplementary Table 2. Multivariable Cox Proportional Hazard Regression Modelling on the Risk of Relapse or Death**

| Model A: | HR | 95% CI | | p-value |
| --- | --- | --- | --- | --- |
| Age at Diagnosis | 1.03 | 1.01 | 1.05 | 0.017 |
| Cardiac involvement | 1.68 | 0.98 | 2.89 | 0.06 |
| Renal involvement | 1.93 | 1.10 | 3.40 | 0.023 |
| Hyperdiploidy, Overall | 1.79 | 1.08 | 2.95 | 0.023 |
| Model B: |  |  |  |  |
| Age at Diagnosis | 1.03 | 1.00 | 1.05 | 0.030 |
| Cardiac involvement | 2.06 | 1.19 | 3.58 | 0.010 |
| Renal involvement | 1.92 | 1.09 | 3.41 | 0.025 |
| Hyperdiploidy, High Risk | 2.12 | 1.16 | 3.87 | 0.014 |

**Supplementary Table 3. Multivariable Cox Proportional Hazard Regression Modelling on the Risk of Death**

| Model A: | HR | 95% CI | | p-value |
| --- | --- | --- | --- | --- |
| Age at Diagnosis | 1.04 | 1.01 | 1.06 | 0.004 |
| Cardiac involvement | 2.10 | 1.13 | 3.92 | 0.019 |
| Hyperdiploidy, Overall | 2.25 | 1.27 | 4.00 | 0.006 |
| Model B: |  |  |  |  |
| Age at Diagnosis | 1.03 | 1.01 | 1.06 | 0.012 |
| Cardiac involvement | 2.70 | 1.41 | 5.18 | 0.003 |
| Hyperdiploidy, High Risk | 3.09 | 1.53 | 6.22 | 0.002 |
